# Accrual Suspensions in Seamless Phase II/III Trials: A Review of NRG Oncology Trials

**DOI:** 10.1200/OA-24-00066

**Published:** 2024-12-10

**Authors:** Chen Hu, Boris Freidlin, Edward L. Korn

**Affiliations:** ^1^ Division of Quantitative Sciences, Sidney Kimmel Comprehensive Cancer Center, Johns Hopkins University School of Medicine, Baltimore, MD; ^2^ NRG Oncology Statistics and Data Management Center, Philadelphia, PA; ^3^ Biometric Research Program, National Cancer Institute, Bethesda, MD

## Abstract

**PURPOSE:**

A phase II/III trial is a type of phase III trial that has embedded in it an intermediate phase II go/no-go decision as to whether to continue the accrual to the phase III sample size. We examine the design characteristics and experience of a well-defined set of National Cancer Institute phase II/III trials, with special emphasis on designed accrual suspensions while awaiting the data to become mature enough for the phase II analysis. This experience is used to highlight the potential of using a calendar backstop to avoid an inordinately long accrual suspension.

**METHODS:**

We identified all phase II/III trials conducted by NRG Oncology or its precursor National Cancer Institute Cooperative Groups (Radiation Therapy Oncology Group, Gynecologic Oncology Group, and National Surgical Adjuvant Breast and Bowel Project). The design characteristics were recorded, and, for completed trials, the trial results in terms of sample sizes and timing of analyses were tabulated.

**RESULTS:**

Twenty-two trials were identified, 14 of which had a time-to-event end point for their phase II component. Thirteen of these 14 trials had designed accrual suspensions. Seven of the eight completed trials had designed accrual suspensions, all of which went on longer than their projected suspension times (3-20 months longer than planned). The trade-offs for using a backstop are discussed using one of these trials as an example.

**CONCLUSION:**

Phase II/III trials with an accrual suspension and a predefined backstop for the phase II analysis can be a useful tool for minimizing patient exposure to ineffective experimental treatments while still obtaining the trial results in a timely fashion.

## INTRODUCTION

Seamless phase II/III randomized clinical trials are phase III trials that have an interim go/no-go decision rule, typically using an intermediate end point for the initial (phase II) component, to decide if the experimental treatment has sufficient activity (compared with the control treatment) to continue the trial to the phase III component.^1^ A phase II/III trial substitutes for a phase II trial followed by a separate phase III trial. Its main advantage over sequential development is the use of the phase II patient data in the phase III analysis, shortening the time to obtain the phase III results and the total number of trial patients treated. The main disadvantage of a phase II/III trial is the commitment to a phase III trial of a specific experimental therapy against a specific control therapy in a specific population (if the phase II results are positive) earlier than if a stand-alone phase II trial had been performed. This could be problematic in a clinical setting with rapid advancements in drug and biomarker development.^2^

**CONTEXT**

**Key Objective**
How frequently does accrual suspension occur in phase II/III oncology trials, and can a calendar backstop help reduce delays in the transition from phase II to phase III?
**Knowledge Generated**
In an analysis of 22 phase II/III trials, 13 trials included designed accrual suspensions and most experienced delays beyond their projected timelines, ranging from 3 to 20 months. The study suggests that a calendar backstop may help manage these delays, allowing trials to progress more efficiently and reducing patient exposure to potentially ineffective treatments. This approach could enhance trial timelines while maintaining the integrity of phase II evaluations.

A key design element of a phase II/III trial is whether to have an accrual suspension while waiting for the phase II data to mature^3^ and its structure if one is used. Phase II/III designs are typically used when sufficient evidence for experimental treatment activity is lacking to directly proceed to a large phase III trial. Therefore, an accrual suspension can be important as otherwise the trial might accrue to almost its full phase III sample size before a potentially negative phase II result is obtained (thus exposing many more patients to a potentially toxic inactive treatment).^4^ For example, NRG-BN007^5^ suspended accrual after 159 patients had been randomly assigned to the phase II part of the trial. The (no-go) phase II decision was made when there were 100 progression-free survival (PFS) events, which occurred 11 months later after the suspension began. If an accrual suspension had not been used in this trial, we estimate that approximately an additional 73 patients would have been accrued to this negative trial (on the basis of its accrual rate). The disadvantage to having an accrual suspension is that it delays getting the phase III results (if the phase II results are positive). It also disrupts the trial process, especially for a multi-institutional trial and if the suspension is long.

There have been studies of the theoretical operating characteristics of phase II/III trials,^6,7^ but this report examines the NRG experience with phase II/III trials, with special focus on the use of accrual suspensions.

## METHODS

We identified by searching the National Cancer Institute (NCI) Clinical Trials Support Unit database for all phase II/III trials conducted by NRG Oncology or its precursor NCI Cooperative Groups: Radiation Therapy Oncology Group (RTOG), Gynecologic Oncology Group (GOG), and National Surgical Adjuvant Breast and Bowel Project (NSABP). For trials with a time-to-event phase II end point, the trial design characteristics for the phase II and phase III components were recorded including the end points, the required sample sizes and numbers of events, the type 1 errors and powers, and the targeted hazard ratios (HRs).

For completed trials with published results, we compared the projected and actual timing of the phase II analysis. The timings were compared in terms of both calendar time and the number of patients accrued. For trials with a designed phase II accrual suspension, we compared the projected and actual phase II accrual suspension calendar times.

## RESULTS

Twenty-two trials were identified. Eight of these trials had non–time-to-event phase II end points and are not considered further here as accrual suspensions would not be relevant, leaving 14 trials (Table 1). The phase II end points were PFS (12 trials), disease-free survival (one trial), and freedom from progression (one trial). The phase III end points were overall survival (OS; nine trials), PFS (one trial), PFS/OS (two trials), metastasis-free survival (one trial), and coprimary end points of PFS and a quality-of-life end point (one trial). The *P* value cutoffs for the go/no-go decisions for the phase II analyses were *P* ≤ .10 (five trials), *P* ≤ .15 (five trials), and *P* ≤ .20 (five trials).

**TABLE 1.** Design Characteristics of Published Phase II/III Trials Conducted by the NRG and Its Precursor Cooperative Groups RTOG, GOG, and NSABP

Trial ID (NCT No.)	Phase II					Phase III					
	End Point	Target HR/Effect Size	Type 1 Error (1-sided), %	Required No. of Events	Power	End Point	Target HR/Effect Size	Type 1 Error (1-sided), %	Sample Size (including phase II)	Required No. of Events^a^	Power, %^b^
GOG-0281^8^ (NCT02101788)	PFS	0.66	20	106	90	PFS	0.66	2.5	250	213	80
GOG-0286B^9^ (NCT02065687)	PFS	0.66	20	60	90	OS	0.66	5.0	540	300	97
NRG-BN007^5^ (NCT04396860)	PFS	0.58	15	100	95	OS	0.72	2.4	485	363	85
NRG-BR002^10^ (NCT02364557)	PFS	0.55	15	69	93	OS	0.67	2.5	360	231	85
NRG-GY005^c^^,11^ (NCT02502266)	PFS	0.625	20	93	90	PFS OS^d^	0.625 0.625	0.83 0.83^d^	340	323^f^ 223	96 90
NRG-GY009^c^^,12^ (NCT02839707)	PFS	0.625	10	110	88	OS^e^ PFS^e^	0.625 0.55	1.24 0.01	320	236 280^f^	91 90
NRG-HN004^13^ (NCT03258554)	PFS	0.65	20	69	80	OS	0.73	2.5	444	364	80
NRG-LU002^14^ (NCT03137771)	PFS	0.60	15	138	95	OS	0.68	2.5	378	278	85

NOTE. The additional six ongoing phase II/III trials were NRG-GU002 (NCT03070886), NRG-GY026 (NCT05256225), NRG-HN005 (NCT03952585), NRG-LU007 (NCT04402788), RTOG-1008 (NCT01220583), and RTOG-1216 (NCT01810913).

Abbreviations: GOG, Gynecologic Oncology Group; HR, hazard ratio; NSABP, National Surgical Adjuvant Breast and Bowel Project; OS, overall survival; PFS, progression-free survival; RTOG, Radiation Therapy Oncology Group.

^a^ If a trial specified the required number of events from only the control arm, the number in this table is given by (1+ HR) times the required number of control arm patients, where HR is the target HR.

^b^ This is the nominal power for the phase III trial not accounting for the phase II analysis.

^c^ This trial had more than one experimental arm. The numbers in this table refer to each experimental arm versus control arm comparison.

^d^ The OS end point was tested only if the PFS end point for that comparison was statistically significant.

^e^ Coprimary end points—both tested.

^f^ The PFS phase III analysis was specified to occur when the OS phase III analysis was performed. This is an estimate of the number of PFS events expected to have occurred at that time.

Among the 14 trials, 13 had designed accrual suspensions, with seven having ≥6-month expected suspensions. Of the eight completed trials with published results (Table 2), seven had designed potential accrual suspensions, with the actual suspensions lasting from 3 to 20 months longer than planned (median 15 months).

**TABLE 2.** Projected and Actual Trial Suspensions for Published Phase II/III Trials Conducted by the NRG, RTOG, GOG, and NSABP

Trial	Timing of Phase II Analysis From Trial Activation (months/patients accrued)				Length of Suspension (from the last phase II patient accrued)		Phase II Result (proceed to phase III or stop)	Phase III Result (positive/negative)
	Projected		Actual		Projected	Actual		
	Months	No.	Months	No.	Months	Months		
GOG-0281	32	176	38	213	NA	NA	Go	Positive
GOG-0286B	17	240	32	244	0^a^	16	Go	Negative
NRG-BN007	23	150	31	159	7	11	Stop	NA
NRG-BR002	42	128	89	129	12	32	Stop	NA
NRG-GY005^b^	22	104	34	106	3	18	Go	Negative
NRG-GY009^b^	33	160	26	160	12	15	Go	Negative
NRG-HN004	24	234	41	186^c^	0	13	Stop	NA
NRG-LU002	45	216	77	218	7	22	Stop	NA

Abbreviations: GOG, Gynecologic Oncology Group; NA, not applicable; NSABP, National Surgical Adjuvant Breast and Bowel Project; RTOG, Radiation Therapy Oncology Group.

^a^ The design was to stop accrual at the 240 phase II sample size if the required number of phase II events was not seen by that time, but it was projected that the required number of events would be seen at that time.

^b^ Sample size for each experimental arm versus control arm comparison.

^c^ After planned interim phase II futility analysis, the trial was closed to accrual.

## DISCUSSION

When the experimental treatment works much better than expected or when the event rate has been overestimated in both treatment arms (the more likely scenario), an accrual suspension time can be much longer than projected. To lessen this problem, a backstop strategy^15^ is recommended: the phase II analysis is performed at the prespecified backstop time if the required number of phase II events has not yet occurred (the backstop length would depend on clinical and feasibility parameters^15^). The downside of using a backstop strategy is that with fewer events (if the backstop analysis is triggered), there is less information about the treatment effect to inform the go/no-go decision rule. Reduced information results in either a decrease in power or an increase in the type I error (or possibly both) of the phase II go/no-go decision rule. To balance these two competing considerations, we suggest using a go/no-go decision rule on the basis of the prespecified cutoff on the hazard ratio scale, rather than a rule on the basis of hypothesis testing with the prespecified type 1 error. The hazard ratio scale cutoff approach results in less power loss relative to the hypothesis testing approach with some inflation of the type I error.

For example, the design of NRG-BR002^10^ had phase II testing at a significance level of 0.15 at 69 PFS events (corresponding to requiring the observed HR to be <0.78) for the trial to continue onto phase III. The trial design had an accrual suspension that was projected to be 12 months, but instead lasted for 32 months awaiting the 69 events. If the trial design had included a backstop of 18 months and the backstop analysis was used (with, say, 38 events), we would have recommended that the HR observed at that time be compared with the designed cutoff HR of 0.78 for the go/no-go decision rather than testing at the 0.15 significance level. Using the 0.78 HR cutoff rather than the 0.15-level testing for an 18-month backstop analysis at 38 PFS events would have resulted in a readout 14 months earlier, at the cost of a power loss from 93% to 86% and an increased type 1 error from 0.15 to 0.22 (for the phase II portion of the trial). In comparison, had the same 18-month backstop analysis been conducted at the same type 1 error of 0.15, the power would have been further reduced to 79% (instead of 86%). If the trial had included a later backstop at 24 months instead of 18 months, and the backstop analysis was applied using the cutoff HR of 0.78 (with, say, 45 events), then the trial would have resulted in a power of 88% (instead of 83% on the basis of 0.15-level testing) and a type 1 error for the phase II portion of the trial of 0.20 (instead of 0.15).

In NRG-LU002,^14^ the phase II testing was designed to detect a PFS HR of 0.60, with a significance level of 0.15 and a 95% power. The go/no-go decision was contingent on whether the observed HR was 0.83 or lower when at least 138 PFS events were observed. Patient accrual, however, was suspended for 22 months—much longer than the projected 7 months—to reach the 138 PFS events. If the trial design had included a 12-month backstop and, say, 115 PFS events had been observed, a go/no-go decision (on the basis of the HR cutoff of 0.83) could have been made 10 months earlier, with a 93% power (compared with the planned 95%) and a significance level of 0.17 (instead of the planned 0.15).

Phase II/III trials with an accrual suspension with a predefined backstop can be a useful tool for minimizing patient exposure to ineffective experimental treatments while still obtaining the trial results in a timely fashion.
